# Prevention of sexually transmitted infections using mobile devices and ubiquitous computing

**DOI:** 10.1186/s12942-015-0010-z

**Published:** 2015-05-03

**Authors:** Felipe Besoain, Antoni Perez-Navarro, Joan A Caylà, Constanza Jacques Aviñó, Patricia García de Olalla

**Affiliations:** Estudis d’Informàtica, Multimèdia i Telecomunicació, Universitat Oberta de Catalunya, Rambla Poblenou 156, Barcelona, Spain; Escuela de Ingeniería en Bioinformática, Universidad de Talca, Avenida Lircay s/n, Talca, Chile; Servei d’Epidemiologia, Agència de Salut Pública de Barcelona, Pça. Lesseps, 1, Barcelona, Spain; CIBER de Epidemiología y Salud Pública (CIBERESP), Barcelona, Spain

## Abstract

**Background:**

Advances in the development of information and communication technologies have facilitated social interrelationships, but also sexual contacts without appropriate preventive measures. In this paper, we will focus on situations in which people use applications to meet sexual partners nearby, which could increase their chance of exposure to sexually transmitted infections (STI). How can we encourage users to adopt preventive measures without violating their privacy or infringing on the character of the application?

**Methods:**

To achieve the goal of preventing STI, we have used the *design and creation* methodology and have developed a prototype software package. This prototype follows the RESTful services principles and has two parts: an Android OS application with emphasis on ubiquitous computing and designed according to General Responsibility Assignment Software Patterns (GRASP), and a server with a web page. To choose the preventive messages, we performed a test in 17 men who have sex with men (MSM).

**Results:**

Our software sends preventive notifications to users when it detects situations such as the activation of particular applications on their smartphones, or their proximity to areas with a high probability of intercourse (*hot zones*). The underlying idea is the same as that for warning messages on cigarette packets, since users read the message just when they are going to smoke. The messages used have been selected from a list that has been rated by the users themselves. The most popular message is “Enjoy sex and enjoy life. Do not expose yourself to HIV”. The user is unaware of the software, which runs in the background.

**Conclusions:**

Ubiquitous computing may be useful for alerting users with preventive and educational messages. The proposed application is non-intrusive because: 1) the users themselves decide to install it and, therefore, users’ privacy rights are preserved; 2) it sends a message that helps users think about taking appropriate preventive measures; and 3) it works in the background without interfering with users unless a trigger situation is detected. Thus, this type of application could become an important tool in the complex task of STI prevention.

## Background

In recent years, there has been a clear increase in the number of outbreaks of infectious diseases that had previously been categorized as being under control [1,2]. One reason could be the fact that globalization and new technologies have facilitated contact, and may have indirectly provoked a large number of infections around the world [3]. In addition, many people, especially young people, perceive these diseases as not being very risky or, simply as having been eradicated. Within these diseases Sexually Transmitted Infections (STI), particularly Human Immunodeficiency Virus (HIV), are especially important. HIV remained one of the world’s principal lethal infectious diseases during the period 2001–2012, as noted by the World Health Organization [4].

The prolific development of information and communication technologies in recent years, including social networks such as Facebook, Twitter, or MySpace, has increased the rate of interrelations within certain population groups [5]. Smartphones have made this process even easier, thanks to software like Grindr [6], ManHunt [7] and Wapa [8], which use georeference techniques to locate partners [9,10] and are readily available through software distribution channels like Google Market for Android and the Apple Store for iOS [11]. Thus, the Internet in general, and smartphones in particular, facilitate meeting sexual partners [12,13]. This scenario promotes *express* dating, which often ends in sexual relationships without appropriate protection, and with regular changes of partner. This situation helps to spread STIs.

To counteract this, health organizations have tried to reduce the transmission of pathologies using IT [13,14]. One such example is “The Just Us Study” [14], which is designed to promote sexual education through the use of an online social network. However, many of these attempts have been limited to personal computers and websites, whereas most of the target population has now migrated from PC’s to smartphones. Therefore, new preventive actions should focus on smartphones.

However, it is not clear whether there is a marked difference between PC-mediated compared to smartphone-mediated contact. PC-mediated contacts are usually made at home and are not *express*, which promotes users into thinking more carefully about their encounters, buying and carrying condoms or other preventive measures, and even cancelling the encounter. In contrast, smartphone-mediated contacts are usually faster [15] since they allow users to find available partners near their current location, promoting *express* dates, and limiting the time available to re-think or prepare the date.

Thus, the question to be addressed in this work is how to make people think of preventive measures when are looking for an encounter that will take place in a few minutes.

To answer this question, we propose to launch alerts and recommendations via users’ smartphones, when they are detected to be looking for an *express* date. These alerts and recommendations will be generated by software that works in the background, without interfering with other applications on the smartphone. These messages will be launched when the system detects that an *express* date is highly probable, just at the appropriate time to help users think about their safety.

This software should meet two important criteria:

1. The use of the software must be voluntary. While it will work in background, users have to install and activate it, and thus it will address only users who think they need to adopt preventive measures to prevent STIs, but who usually forget.
2. The software should not criminalize behaviors or sexual options, but rather should seek to make them safer.

The key is then to determine how a smartphone could “detect” when an *express* date is about to occur? To address this, we propose that two kind of events could trigger the launch of a message: the execution of a contact application, and proximity to a place where *express* dates usually take place, such as some hotels, saunas or other locations, which we will call *hot zones*.

Thus, in this research we seek to use smartphones to promote the use of condoms and other preventive measures in *express* dates, and to reduce risky sexual practices. The result is an Android application that uses ubiquitous computing to launch preventive messages when users are close to the date.

It is important to note that: 1) prevention of STIs is multidimensional and many distinct approximations are required; and 2) STIs are often asymptomatic and require constant surveillance and prevention to make people aware of them. Therefore, the solution proposed in this paper deals with only one of these dimensions.

The paper is structured as follows: first, the methods used to create the application are introduced, and the architecture and use cases of the application are shown; we then present the results, which are mainly the main features of the application and its utility as a preventive tool; finally, we describe our conclusions and future work.

## Methods

The methods used in this research follow the design and creation approach to create a georeferenced application that uses ubiquitous computing concepts to prevent contagions. To choose the preventive messages, we performed a test in 17 MSM. The results of this test are shown in Results and discussion section.

In this section, we describe the use cases to which the application will respond, its architecture, and its key components and their functions.

### Use cases

To understand how the software behaves, we present two general use cases. These use cases briefly explain each process and its interaction with the various solution components, which will also be outlined in this section. The first use case triggers messages when a contact application is launched on the smartphone; the second triggers a message when the application detects that users are within a *hot zones*, as defined above. Use case: *General detection of a contact application (such as ManHunt)*. Actor: User, Information server. Purpose: Detect a contact application at an appropriate time and notify users of preventive measures and, if they request it, information on the nearest place to get them. Summary: This use case begins when the notification service detects that a contact application has been executed. The software launches a preventive message (see Results and discussion section). Preconditions: The notification service runs independently of how it was started, for example, by software or by the operating system (it is assumed that users have previously configured the software).

Use case: *General detection of* hot zones Actor(s): User, Information server. Purpose: Detect a *hot zone* at an appropriate time and notify the user of its location. Summary: This use case begins when the notification service detects a *hot zone* near the user’s current position. The alert is communicated using a RestFul service to a server containing a database of *hot zones*. Then, the software notifies the user about the zone. Preconditions: 1) The database of *hot zones* should contain information on their positions and radius; 2) users should have launched the notification service within the software (it is assumed that users have previously configured the software).

More details on use cases *General detection of a contact application* and *General detection of* hot zones are provided in Tables 1 and 2, respectively.

**Table 1:** **General detection of a** ***hot zone***

**Actor’s actions**	**System’s answers**
	1.- This use case starts when the*notification service* obtains users’geoposition.
	2.- The notification service requestsinformation from the server withusers’ geoposition and the *hotzones* closest to them.
	3.- It determines if users are near a*hot zone*, using distances that havebeen configured previously.
	4.- It notifies users of the *hot zone*through the notifications bar.
5.- Users select thenotification.	
	6.- The system opens a windowusing *mapview*, in which it shows the various *hot zones* including their radius.
7.- Users can select arisk presented in themapview and obtaininformation about it(available when theapplication is usedfor infectious diseasesprevention).	
**Alternative flow:**	3.- The *notification service* did notfind a *hot zone* near users’geoposition, therefore the system checks again after X amount of time has passed or Y meters have beentraveled.

This table presents an extension of the *General detection of a geographic use case*, in a conversational format, which emphasizes the interaction between the actors and the system.

**Table 2:** **General detection of a monitorized application**

**Actor’s actions**	**System’s answers**
1.- This use case startswhen users start arisk-associated software.	
	2.- The *surveillance service* detectsthe launching of a contactapplication.
	3.- It notifies users of the launch ofthe application through thenotifications bar
4. - Users select thenotification.	
	5. - The system opens a window with mapview in which the nearest medical center to his/her position is shown.
6. - Users can select thehealth center or pharmacyand obtain informationabout it.	
7.- Users can select a riskpresented in the mapviewand obtain informationabout it.	
**Alternative flow:**	2.- The *surveillance service* did not find a risk-associated application, therefore the system checks again after X amount of time.

This table presents an extension of the *General detection of a monitorized application*, in a conversational format, which emphasizes the interaction between the actors and the system.

As evident from these use cases, our proposed solution requires us to consider the following aspects:

- Creation and storage of geodata sources.
- Development of a web service for distributed systems.
- Integration with mobile devices.

In the following subsection, we propose an architecture that takes these three items into account

### Architecture of the solution

To implement a distributed system that is scalable and independent, we used RESTful services principles in conjunction with various open source components [16,17]. REST defines a set of architectural principles focusing on a system’s resources, all the various states of the resources are addressed and transferred using the HTTP protocol, which allows the system to interact with any client capable of implementing the HTTP protocol.

**Figure 1:**
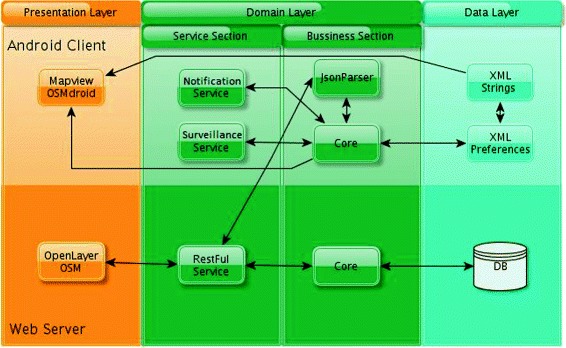
Implemented architecture and essential components and services. The architecture consists of three main layers: the presentation, domain and data layers, representing the interaction between the essential components of the solution. This representation is divided between the Android client (top) and the web service (bottom). The re-used open source components in the proposed architecture are also shown.
- The mobile component: this is mainly an Android [18] client.

Figure 1 shows the conceptual architecture that integrates all components and services of our solution, according to the REST principles. This system has two components:

- The web component: this is mainly a web server for managing the application data, and includes a web client;

### The web component

The web component allows us to save and manage geographic information of locations and zones that are relevant for STI prevention: for example, places where users can acquire condoms, or zones where contacts usually take place. Health professionals can create and update this information to optimize preventive actions.

From an architecture point of view, the web component has three layers: the presentation layer, the domain layer and the data layer.

#### The presentation layer of the web component

**Figure 2:**
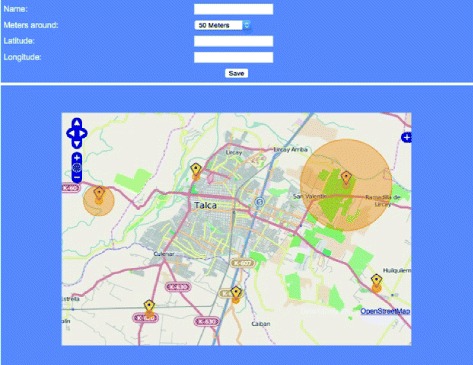
Web client: The user interface of the web server has been constructed by integrating OpenLayers and OpenStreeMap such that users can visualize the various alerts that have been included in the database. New alerts can be created by georeferencing through the map and mouse interaction, and relevant information can be added to the alert. (Labels translated from Spanish).

The presentation layer of the web component corresponds to the web site that allows us to introduce georeferenced locations and zones (see Figure 2). PHP has been used as a control language [19], OpenStreetMap (OSM) [20] as a free map framework, and the OpenLayers libraries [21] to handle the various layers of the maps. Note that while OpenStreetMaps (OSM) can be used as a source for points of interest, these points do not currently contain the specific information required for the proposed preventive application. Therefore, OSM is used as base cartography, and to identify the location of the elements.

#### The domain layer of the web component

The domain layer is subdivided in two parts: the service section and the business section.

##### The service section in the web part.

The service section implements a RESTful service that processes requests and communicates with the business section. Note that this is the section that ultimately interacts with clients associated with the service. This architecture provides a solution with greater interoperability and modularity. Therefore, the architecture will not be affected by eventual transformations on the server or client side.

Usually, client applications request resources from the server using GET methods from the HTTP protocol. These resources are identified using a URI. The HTTP protocol is used in RESTful services to interact with the resources of the server. This is very beneficial for mobile clients because of their limited resources. Using this design, the client load will be lightened by turning it over to the server, which has more resources.

The RESTful service receives a request from the Android client (*notification service*), which also sends the user’s geolocation and a search radius. The request is a simple URI.

Using this information, the RESTful service communicates with the persistent data layer on the server (see Figure 1). More specifically, it communicates with the database in order to verify if users are near a relevant area. If this is the case, the service returns the area in order to display it on the mobile device.

##### The business section of the web domain layer.

The business section corresponds to the core of the application, which executes a geopoints search algorithm that interacts directly with the data layer.

#### The data layer of the web component

The data layer (or persistence data layer), is a database that stores geopoint data, as well as their associated characteristics, and information added by health professionals.

### The mobile component (Android client)

A key element of the application is the interaction with users through the Android client. With the aim of developing Android software with reusable, modular and independent components, development was based on General Responsibility Assignment Software Patterns (GRASP). These patterns allow us to create high cohesion and low coupling between objects [22,23].

The Android client is also divided into the same three layers as the server, namely the presentation, domain and data layers.

#### The presentation layer of the Android client

The presentation layer contains two main parts: the interface for accessing data on the configuration, and mode of execution; and the integration of OpenStreetMap with Android, which displays features in a particular area.

We used the *cl.tfm.Maps* package, which contains the classes required to visualize *hot zones*, using the OSMDroid libraries [24].

The map obtains the stored preferences, such as the search radius for *hot zones*, instantiating a *PreferenceManager* object in the Android API. The minimum search distance was selected so that users would have enough time to respond and take preventive action. Using these data, we can instantiate the class that communicates with the *RESTful* service, *JsonParser*. This class sends a *request* with a URI containing the longitude and latitude of the mobile device’s current position, and the radius of the search. The response is saved in an *ArrayList* of the *OverlayItem* type, which is defined in the OSMdroid[24] API. Inside each list, each OverlayItem object contains the latitude, longitude, affected radius, and the icon to be drawn on the map. Since this functionality can help to prevent other localized diseases, it also can give information about the risk found, although we will not address this functionality in this paper.

**Figure 3:**
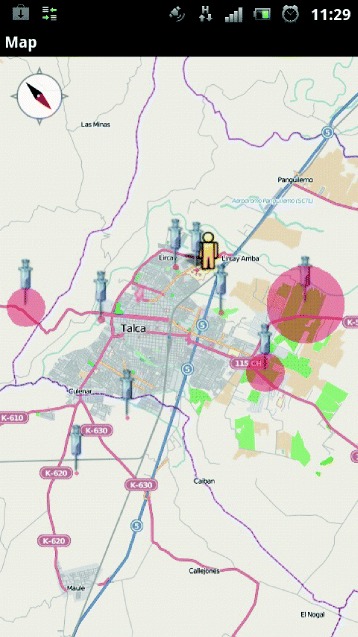
User Interface, Android client. The interface shows the various nearby *hot zones* (red circles) and users can select these areas to obtain more information. (Labels translated from Spanish).

In order to visualize the *hot zones* and health points (see Figure 3), we have created an object instance of the *Activity* type [18], which follows the life cycle of an *Activity*, defined by the Android operating system. To draw on the map, we constructed three layers:

- Map layer or *mapview*: In the main Activity, specifically in its *onCreate()* state, we load a map view, which is defined by an XML file. We made the layer and added several features defined by the OSMdroid API, such as *setBuiltInZoomControls(); enableCompass(); enableFollowLocation();*.
- Point layer: This obtains the latitude, longitude and icon from the *ArrayList* created by the JsonParser object, and adds OverlayItem objects to the layer.
- Area layer: A new Overlay object was instantiated, overriding the *draw()* method. In this method, we review the *ArrayList of the OverlayItem type* list with the geopoints. We also create a point at the same location but with a circle drawn in the middle using *Canvas*, specifically with *drawCircle()*. In order to measure the radius accurately, we obtain the projection of the map and we transform it using the geopoint. Similarly, to transform the distance in meters, we use the API method *metersToEquatorPixels()* from OSMdroid. Thus, each time a ZoomIn or ZoomOut is performed, the method will re-draw the radius of the circle according to the map’s projection and Zoom.

Each *Activity* in Android has its own life cycle. Therefore, it is important to point out that each state in the cycle interacts with the operating system. We worked with the following states: *onCreate()*, *onStart()*, *onDestroy()*, in which different interactions with the respective components of the operating system were instantiated, run and killed.

#### The domain layer of the Android client

As for the web component, the domain layer is divided in two sections: the service section and the business section.

##### The service section of the client domain layer.

The service section implements two internal monitoring services that are responsible for reviewing and comparing certain mobile states related to the location and the applications that are running that time. Simultaneously, it interacts with the business section.

There are two kind of services: the notification service and the surveillance service. *The notification service in the client domain layer*

The notification service sends the user’s geolocation and the search radius to the RESTful service on the server via the business section. With this information, the RESTful service communicates with the server’s persistent data layer (see Figure 1). More specifically, it communicates with the database in order to verify if the user is near a relevant area. If this is the case, the service returns the area in order to show it in the mobile device. Thus, this service detects *hot zones*. From technological point of view, it is linked with two essential components: *JsonParser* and *NotificationManager*. The algorithm for detecting if we are near an area stored in the web server database is based on the implementation of a *LocationListener*.

Once the service is initialized, in the *onCreate()* state, we define the following resources:

1. *LocationManager*
2. *NETWORK_PROVIDER*
3. *GPS_PROVIDER*
4. *NotificationManager*

The descriptions of these resources are available in the documentation of Android API [18].

We have two options to geolocate users: *GPS_PROVIDER*, that uses GPS; and *NETWORK_PROVIDER*, that uses the network. *GPS_PROVIDER* is more accurate; however, it uses more battery. In contrast, *NETWORK_PROVIDER* is less accurate, but it uses less battery. In the case of our software, the accuracy of the *NETWORK_PROVIDER* is sufficient because it has a noise which is a short distance by foot. Therefore, we ultimately decided to use the *NETWORK_PROVIDER* to save the battery.

Once the provider is selected, we call a *requestLocationUpdates()*, defining its parameters: the provider, the quantity of time and the distance that we want to update.

After the update, if the location has changed the *onLocationChanged()* method, we get the current position, passing it to the notification class. This instantiates JsonParser with the current location of the mobile with the aim of getting a *hot zone* near the radius previously defined by users.

If there is a message, it is communicated with the operating system’s *NotificationManager*. To alert users, we have developed sound and vibration notifications, using the message bar of the mobile device to display a preventive message. In addition, an *Intent* [18] is activated when users select the message in the bar. If there is no message to send, the service will continue running until the user or the operating system kills the service.

*The surveillance service in the client domain layer.* The surveillance service monitors the launch of some applications, such as when users run a program to find sexual partners. When this happens, the service sends the user’s geolocation, in an URI, to the RESTful service on the server, via the business section. With this information, the RESTful service communicates with the server’s persistent data layer (see Figure 1). Specifically, it communicates with the database to get all health points within the chosen radius where the user can obtain condoms or other preventive measures, and returns their geopoints and names. Note that in some situations the service might not find any response within the given radius.

When any of the services detect a situation that requires a notification, this message is sent to user. The *cl.tfm.services* package stores the internal services that are responsible for notifying users when they run a monitored program that facilitates contacts, or when the user is near to a *hot zone*.

##### The business section of the client domain layer.

The business section contains the core of the application that interacts with *JSonParser*, and is responsible for sending messages from the notification and surveillance services to the RESTful service of the web component.

The *cl.tfm.JsonParser* package has the classes required make different *requests* to the *RESTful* service and process data, thereby facilitating their subsequent representation by other components of the software.

#### The data layer of the Android client

The data layer corresponds to the application’s preference settings, and also contains the strings associated with the application, which are independent of all the other layers.

### Ethics and consent statement

We did not have to ask for ethical approval because at the time of the study, it was not legally necessary to ask for ethical approval for a study where no health-relevant information and no personal data was collected from participants.

## Results and discussion

In this section, we will discuss the results, showing how users interact with the software and afterwards, in the Conclusions section, we will draw several conclusions regarding how ubiquitous computing can be used as a preventive tool.

When users run an application such as *Grindr* or *Wapa* to look for sexual partners, they are not simultaneously informed about risk of transmission of STIs. While users are likely aware of this possibility, they may forget about it when they use this kind of application. In addition, mobiles allow users to rendezvous shortly after making contact, which can give them little time to remember the recommended precautions. Thus, it would be interesting to be able to remind users of what they already know, by launching a preventive message at precisely the moment when it would be most relevant and would have the greatest impact [12,25].

An example of this idea is the use of shocking images on cigarette packets to reflect the risk of smoking, such as a photo of yellow teeth or a cancer patient. Thus, when smokers reach for a cigarette, they receive a visually powerful preventive message. Several studies ([26-28]) that have analyzed the impact of this type of message conclude that they are important for the complex task of changing behaviors. While this comparison with cigarette packets is interesting, the problem addressed in this paper is different because: 1) in this case the action itself is not a risk, but rather the failure to use preventive measures when performing the action; and 2) users are told to do something, rather than not to do something. It is also important to note that potential users of the application are people that are already concerned with their sexual health, and therefore such messages might be more likely to have a positive effect.

**Figure 4:**
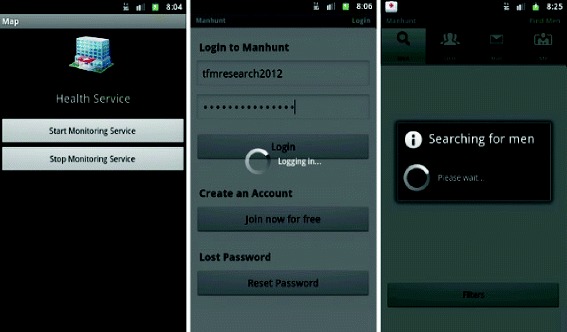
User Interface, Android client. The software runs in the background and alerts users when certain applications are run. (Labels translated from Spanish).

As described above, the situations that can trigger the message are:

- *Execution of some kind of application*, which is the primary function and applies in situations where users open applications designed for contacting sexual partners, such as *ManHunt* (see Figure 4).
- *Proximity to a geographical zone where sexual contacts often take place*, which is the secondary function and applies in situations where a user enters or is near to a *hot zone*.

It is important to note that the application not only sends a message, but also shows a map with the route to the nearest point where they can obtain preventive measures.

Once the notification has been displayed, the software can interact with other installed applications, for example allowing the user to share the notification through email, text message, social networks, etc. Thus, users have a fully-connected experience that can also help promote prevention among others. When they receive a notification, the user can make an informed decision regarding the possible consequences of their behavior. As a result, the use of this software raises users’ awareness of their actions and encourage them to take steps to limit the spread of STIs.

In addition, the proposed software could also offer an effective means of prevention in some cities, by helping users avoid certain areas when not carrying appropriate preventive measures. Therefore, as a measure for mitigating transmission of diseases, this software gives users access to information about contact areas. When users open the software, all the *hot zones* close to users geolocation are retrieved and shown on the map screen (see Figure 3). Each red circle corresponds to a *hot zone*, and the user can obtain information about each *hot zone* by touching the icon associated with it. Therefore, as a measure for mitigating transmission of disease, this software gives users access to information about contact areas.

It is important to note that, although in the present paper the application was addressed to MSM, such an application could also be potentially useful for sexually active men (SAM), including bisexuals.

In the following subsections, we introduce three key aspects for the effectiveness of the application: the levels of functioning (conscious and automatic); the notifications launched; and the interface.

### Levels of functioning

This application operates in two modes that reflect its function at the automatic and the conscious level:

- **Automatic level (AL)**: This option must be configured in the software. Users install the application if they decide that they want to receive a preventive message when they are likely to have a *express* date. Once installed the application runs constantly in the background, so users do not need to run it in order to receive notifications. This gives users maximum liberty in using their mobile, while keeping them informed. Note, however, that it must be the user who voluntary decides install and use the application.
  When users run a service, even if the application is closed, the service will continue to run in the background. This allows users to have a completely different experience, since they will be able to use their device normally: to talk on the phone, navigate the web, or simply leave them idle.
  Running the surveillance service to monitor a contact application or selecting the ubiquitous mode provides constant monitoring of the list of programs that are running in the operating system (this service checks the program list every 3 minutes).
- **Conscious level (CL)**: In this mode, users run the application with the purpose of finding zones identified as having a high probability of sexual contacts, the so-called *hot zones*. Note that this function is location-dependent: in some cities there are locations where contacts are more likely, while in large cities like Barcelona, with more than 7 Million tourists per year, mobility is very high and it may be difficult to identify specific locations where contacts take place. Even so, in Barcelona there are some specific points where sexual contacts are more likely, like some clubs or gay saunas, and therefore, this functionality could also be useful.

The omnipresence and imperceptibility of this preventive notification service is a defining characteristic of ubiquitous computing [14,29]. The main advantage of this ubiquitous system is that the user is not conscious of the fact that their smartphone is being used as a healthcare device.

### Preventive messages

The notifications launched play a key role in prevention objective. Studies in adolescents show that the message itself is important [30-32], as is the time when it is sent, and its format [33]. In all of these studies, however, messaging service is run by an operator who sends the message. In some cases, teenagers have the opportunity to answer the message[34] and it is common for these services to have a face-to-face component. In other examples based on Public Service Announcements [32] the messages are longer and more complex than the short message used on a smartphone.

Our proposed software is an automated system of unattended messaging, so it is even more important to carefully choose the messages previously. In this sense, these messages are more similar to those used on cigarrete packets, where impact reviews demonstrate the importance of clarity of the message [35].

To choose the preventive messages, as said before, we performed a test in 17 MSM, since they are at high risk of infection with HIV. Their demographic characteristics are shown in Table 3. Nearly 60% are in their thirties and more than 20% are over 50 years of age. Most were Spanish (11), and 6 were from other countries.

**Table 3:** **Demographic characteristics of volunteers who helped to choose the messages (** ***n*** **=17)**

**Age**		**Origin**	
21–30	17.6%	Spain	64.7%
31–40	47.1%	Andorra	5.9%
41–50	11.8%	Brazil	5.9%
51–60	23.5%	Chile	5.9%
		Honduras	5.9%
		Peru	5.9%
		Dominican Republic	5.9%

This table shows the demographic characteristics of people who helped to choose the messages.

Regarding the places where volunteers find a partner (Table 4), 78.1% go to specific locations (saunas, discotheques, bars, gymnasiums and parks), 21.9% use geolocators and 28.9% use the internet^a^. Therefore, our software could be useful to most of these volunteers because they find partners in specific locations, or use geolocator applications.

**Table 4:** **Places where users usually find partner (** ***n*** **=17)**

**Place**	
Gay sauna	28.1%
Internet	28.1%
Geolocators	21.9%
Discoteques	21.9%
Bars	21.9%
Has partner	3.1%
Gimnasium	3.1%
Beach	3.1%
Park	3.1%
Street	3.1%

Percentages does not sum 100% because every volunteer could mark several options.

Nonetheless, while we perceived all participants as potential users, only 56.3% considered themselves or people like them as the target of the application (see Table 5), while the remaining 43.8% considered that this kind of application targets people different from them. Regarding age, the most popular answer (30.6%) considered that this application targets people aged 21–30 years, which corresponds to the age of most of the volunteers, and only 19.4% considered that it targets people of any age. When asked if they would download the application, 72% answered affirmatively.

**Table 5:** **Target of the application according to potential users’ perception (** ***n*** **=17)**

**Target profile**		**Target age**	
Anybody	43.8%	15–20	16.7%
Someone like me	12.5%	21–30	30.6%
Someone different from me	43.8%	31–40	19.4%
		41–50	11.1%
		51–65	2.8%
		Any age	19.4%

This table describes the volunteers’ perception as the target of the application, from the profile point of view, as well as from the age point of view. Percentages do not sum to 100% because each volunteer could mark several options.

Volunteers were asked to choose the messages they found most and least suitable as preventive messages, and were also offered the chance to propose new messages. The most popular sentences, which will be displayed randomly by the application, were as follows:^b^

- Enjoy sex and enjoy life. Do not expose yourself to HIV
- Remember: HIV is invisible
- Appearances can be deceiving
- Take the test, avoid AIDS, condoms please, avoid HIV

In contrast, the least popular sentences were:^c^

- Every year 700 people become infected with HIV in Catalonia
- Every treatment of HIV costs 12,000 €/year
- 2 ×1! HIV is free!
- Packaging, where are you going? Where the condoms go?^d^

### Interface

Our objective was to develop a simple interface where users could access information quickly and configure the software, including minimal functionalities, such as ZoomIn and ZoomOut, to more complex functions, such as seeing different locations on the map.

**Figure 5:**
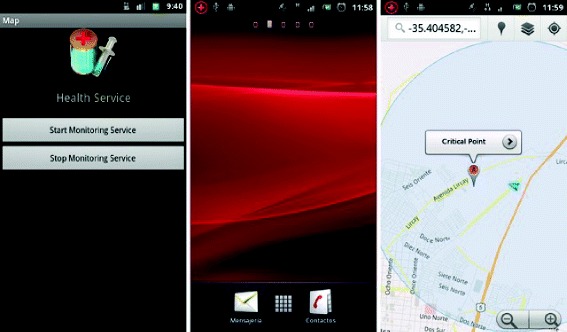
Notification service of *hot zones*. This sequence shows appears when users launch the notification service is running, and the service detects a *hot zone* near the user’s location of users. The service also notifies users in the message bar. When users select that notification, the software opens a new window with the location of the *hot zone* and the users. (Labels translated from Spanish).

The monitoring services work according to the user’s configuration. Figure 5 shows how users can manually turn on the service. The appearance of the message prompts users to touch the screen, which opens map visualization software installed on his or her mobile, such as OSMdroid or Google Maps, showing their current position and the area of interest.

The software also has some features that can be customized by users via the data configuration screen, under the *Android Guidelines*:

- Show instructions (CL and AL): Shows a brief summary of how the software works.
- Ubiquitous mode (AL): Initializes the notification service whenever the mobile device is switched on, without users needing to start the process every time.
- Vibration (AL): Sets the mobile to vibrate when an alert is shown.
- Sound (AL): Sets the mobile to make a sound when an alert is shown.
- Radius distance (CL): Sets the search radius for *hot zones*.
- Monitoring time (CL): Defines how often users want the services to monitor *hot zones*.

The parentheses indicate to which level corresponds every item of configuration, conscious or automatic.

## Conclusions

The aim of this research was to investigate how ubiquitous computing could be useful in preventive health. There is a need to develop new preventive health methods, and we focused on georeferenced ubiquitous computing with mobile devices because of the popularity of these devices and their connectivity with the environment.

Although we live in a society where it is easy to access information, there are still people who are not aware of STIs and how they spread as a consequence of not taking appropriate preventive measures. In addition, mobile devices and applications now allow users to meet nearby partners and have *express* dates, giving them little time to think about safety measures. In this paper we have proposed an application that provides users with information that promotes safe sex.

The Android application we have developed sends a preventive message when the user: 1) runs a contact application; or 2) approaches a zone where sexual contacts are known to take place regularly (*hot zone*). Moreover, a module of this application helps the user find places where they can acquire preventive measures.

The application works in background and users are unaware that it is running (it runs at an automatic level). However, users have to install the application, i.e. its use is voluntary. The target of the application are, therefore, people who are already conscious about STIs but forget about safety measures when using mobile devices to meet partners. The messages displayed do not try to prevent the behavior itself, but rather to inform the user of preventive measures that should be taken into account. The final decision is then up to the user.

In addition, there is another functioning mode called the *conscious level*, which allows users to detect *hot zones* and avoid them. This functionality could also be used to find and go to these zones, although, since the software targets people who are concerned about their sexual health, we do not expect this to be its main use.

We believe that this type of application could help to reduce the high incidence of STIs, including HIV infection. The main advantages of this application is: 1) currently, it is common for people to use mobile devices for dating, so these devices are the most direct platform from which to launch preventive messages; 2) the preventive messages arrive at the most appropriate time, when the sexual contact is imminent, so that users are alerted at their moment of greatest vulnerability; and 3) users install the application voluntarily so we do not expect it to be perceived as intrusive, which is one of the main complaints of the target group.

Finally, it is important to note that the addition of new functionalities in this prototype is technically straightforward to implement, given that it has a modular architecture with high cohesion and low coupling between classes.

Further work will be centered on measuring the real impact of this proposal in terms of statistically significant study groups with the goal of improving technologies, interfaces and interaction with users, as well as acceptance of the software by users and the real impact of the application. Another challenge, especially in the context of the secondary function for *hot zones*, will be to integrate with world data banks such as *HealthMap* [36] to distribute more georeferenced information in the field of health.

## Endnotes

^a^ The sum of the percentages is not 100% because some volunteers used more than one of these channels.

^b^ Translated from Catalan.

^c^ Translated from Catalan.

^d^ Wordplay in Catalan. Untranslatable.
